# Strategies to prevent seroma formation after mastectomy surgery: A systematic review and meta-analysis

**DOI:** 10.1016/j.jpra.2025.11.021

**Published:** 2025-12-01

**Authors:** Abdulrahman Makhseed, Sara Alneamah, Mark Mofid Atnasious Abdelmaseeh, Sarah Al youha

**Affiliations:** a Department of Plastic Surgery, Jaber al Ahmad Hospital, Ministry of Health, Kuwait; b faculty of Medicine, Assiut University; c Plastic Surgery, Kuwait University

**Keywords:** Seroma, Mastectomy, Flap fixation, Quilting sutures, Surgical complications, Systematic review, Meta-analysis

## Abstract

**Background:**

Seroma formation is a frequent and challenging complication after mastectomy surgery, with incidence rates ranging from 3 % to over 85 % (1). Despite numerous proposed interventions, no universally accepted standard for prevention exists.

**Methods:**

A systematic review and meta-analysis was conducted following PRISMA guidelines. PubMed was searched for randomized controlled trials, cohort studies, and systematic reviews published in the past 10 years that evaluated interventions to prevent seroma after mastectomy surgery. Thirty-four studies met inclusion criteria. Data were extracted on study design, intervention, comparator, seroma incidence, seroma volume, and statistical outcomes. Pooled odds ratios (OR) and 95 % confidence intervals (CI) were calculated for each intervention group using a random-effects model. Heterogeneity was assessed with the I² statistic.

**Results:**

Quilting sutures demonstrated the greatest efficacy, reducing seroma incidence by 76 % compared to standard closure (pooled OR 0.24, 95 % CI: 0.11–0.52, I² = 0 %). Flap fixation also significantly reduced seroma rates (pooled OR 0.43, 95 % CI: 0.26–0.70, I² = 0 %). Steroids and selected adjuncts (OK-432, hemostatic powders, diuretics) were associated with a 59 % reduction in seroma incidence (pooled OR 0.41, 95 % CI: 0.26–0.64, I² = 36 %). Drain management strategies showed that early or no drain removal may increase seroma risk (pooled OR 1.99, 95 % CI: 0.96–4.14, I² = 54 %), though not statistically significant. Tissue adhesives (TissuGlu®, fibrin glue, cyanoacrylate) and harmonic scalpel showed modest or inconsistent benefits (adhesives pooled OR 0.63, 95 % CI: 0.35–1.14, I² = 57 %; harmonic scalpel pooled OR 0.59, 95 % CI: 0.36–0.96, I² = 0 %). Nitroglycerin ointment did not significantly reduce seroma formation (pooled OR 0.78, 95 % CI: 0.31–1.96, I² = 0 %).

**Conclusions:**

Quilting sutures and flap fixation are the most effective interventions for preventing seroma after mastectomy surgery, with strong supporting evidence and minimal heterogeneity. Output-based drain management and selective use of steroids or adjuncts may further reduce risk in high-risk patients. Routine use of tissue adhesives, harmonic scalpel, or nitroglycerin ointment is not supported by current evidence. Future research should focus on standardized outcome reporting and direct comparisons of effective strategies, particularly in the setting of mastectomy reconstruction.

## Introduction

Seroma formation remains one of the most common postoperative complications following mastectomy and immediate reconstruction, with reported incidence rates ranging from 3 % to over 85 % depending on surgical technique and patient factors.^1^ Despite its prevalence, the management and prevention of seromas remains variable across institutions, and no universally adopted standard of care exists. Conventional strategies such as closed suction drainage, quilting sutures, and flap fixation are routinely employed, yet these approaches differ in timing, technique, and duration from center to center.

This inconsistency in practice reflects the lack of high-certainty evidence supporting any single superior method of seroma prevention. Moreover, innovation in this field has stagnated, with few novel interventions gaining widespread adoption in recent years. Given the impact of seromas on patient recovery, surgical outcomes, and healthcare costs, there is a clear need to reassess both established and emerging preventative strategies.

The aim of this systematic review is to evaluate the current landscape of seroma prevention following mastectomy surgery. Through critical appraisal of clinical studies, we aim to identify techniques with strong evidence of efficacy and highlight areas where further research and innovation are warranted.

## Methods

### Search strategy

A comprehensive literature search was conducted using PubMed to identify relevant clinical studies evaluating interventions aimed at preventing seroma formation following mastectomy surgery. The search strategy combined key terms related to the condition, surgical context, and preventive techniques as follows:

(“Seroma”[Title/Abstract] OR “Seroma formation”[Title/Abstract]) AND (“Breast surgery”[Title/Abstract] OR “Mastectomy”[Title/Abstract] OR “Breast reconstruction”[Title/Abstract] OR “Reduction mammaplasty”[Title/Abstract]) AND (“Prevention”[Title/Abstract] OR “Quilting sutures”[Title/Abstract] OR “Fibrin sealant”[Title/Abstract] OR “Drains”[Title/Abstract] OR “Drainage”[Title/Abstract] OR “Surgical techniques”[Title/Abstract]).

The search was limited to articles published in English within the past 10 years. Eligible study designs included randomized controlled trials (RCTs), prospective or retrospective cohort studies, systematic reviews, and meta-analyses. Only studies that explicitly evaluated interventions aimed at reducing the incidence or volume of seroma formation after breast surgery were included. Studies focusing on other postoperative complications, or those involving surgeries unrelated to the breast, were excluded.

The final selection comprised 34 studies that met the inclusion criteria and were included for qualitative and quantitative synthesis.

### Eligibility criteria

Studies were eligible for inclusion if they evaluated an intervention intended to prevent seroma formation following mastectomy surgery and reported measurable clinical outcomes, such as seroma incidence or volume. Only original research articles published in English within the past 10 years were considered. Eligible study designs included randomized controlled trials, prospective clinical trials, and systematic reviews that compared an intervention against a control or standard technique.

We excluded studies that were retrospective in design, case reports, expert opinions, or letters to the editor. Articles focusing exclusively on cosmetic procedures, radiotherapy, or brachytherapy without addressing seroma prevention were also excluded.

### Data extraction

Relevant data from each eligible study were independently extracted by the primary author using a standardized extraction sheet. The following information was recorded for each study: first author, year of publication, study design, type of intervention, comparator, number of patients in the intervention and control groups, incidence of seroma formation in each group, and, when available, the mean volume of seroma, standard deviation, *p*-values, odds ratios (OR), relative risks (RR), and 95 % confidence intervals.

In cases where multiple outcome measures were reported, preference was given to the incidence of seroma and volume at 3 weeks postoperatively, as this was the most consistently reported time point across studies. For studies reporting data in a non-standard format (e.g., medians or interquartile ranges), data were included qualitatively if conversion was not feasible. Discrepancies or unclear values were resolved by reviewing the full text of the original articles.

Data were stratified by type of intervention (e.g., quilting sutures, drain management, flap fixation, topical agents) to allow for subgroup analysis and pooled effect estimates.

### Quality assessment

The methodological quality of included studies was assessed independently by two reviewers. Randomized controlled trials were evaluated using the Cochrane Risk of Bias tool (RoB 2), which assesses seven domains including randomization process, deviations from intended interventions, missing outcome data, measurement of the outcome, and selection of the reported result. Each domain was rated as having a low, unclear, or high risk of bias.

Non-randomized studies, including observational cohort and case-control designs, were assessed using the Newcastle–Ottawa Scale (NOS), which considers study selection, comparability of cohorts, and the ascertainment of outcomes. Studies scoring six or more out of nine points on the NOS were considered high quality.

Discrepancies between reviewers were resolved by consensus or by consultation with a third reviewer.

### Statistical analysis

Statistical analysis was performed to calculate pooled odds ratios (ORs) with 95 % confidence intervals (CIs) for each intervention group. Separate forest plots were generated to visually represent the effect sizes and weight of each included study. A random-effects model (Mantel-Haenszel method) was used to account for between-study variability.

Heterogeneity was assessed using the I² statistic, with values of 25 %, 50 %, and 75 % interpreted as low, moderate, and high heterogeneity, respectively. A chi-square (χ²) test was also used, with a *p*-value < 0.10 considered indicative of significant heterogeneity.

For intervention groups with at least 10 studies, publication bias was assessed using funnel plots and Egger’s regression test, with a *p*-value < 0.05 considered evidence of small-study effects.

All statistical tests were interpreted at a 95 % confidence level, and complete numerical data were available for all pooled analyses.

## Results

### Study selection

**Figure 1:**
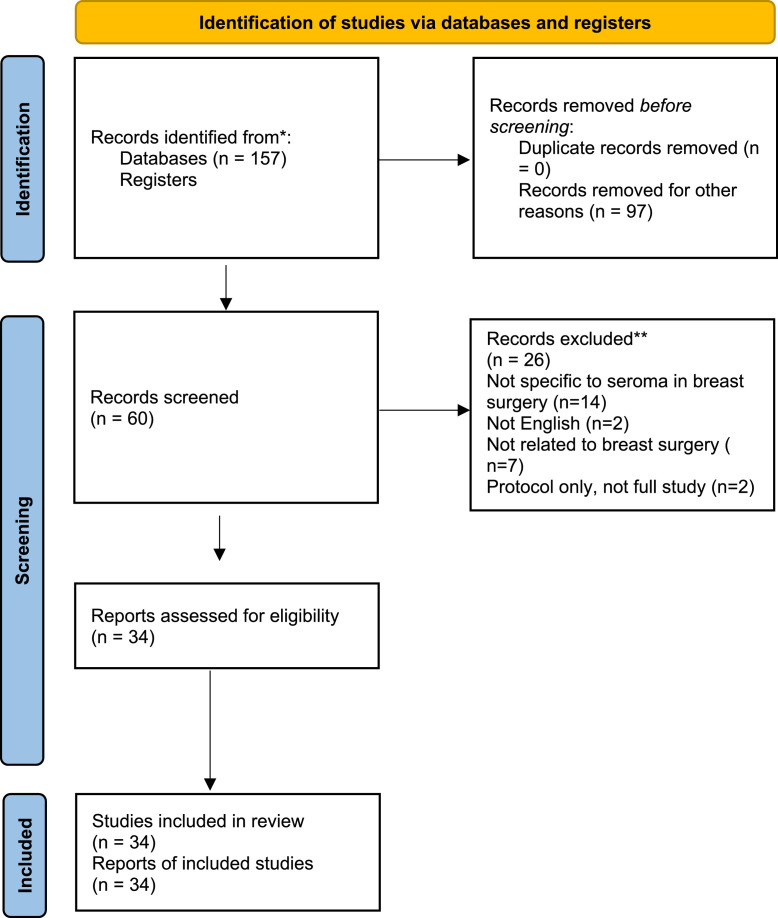
Preferred reporting items for systematic reviews and meta- analysis (PRISMA) flow diagram of literature screening and selection.

The PRISMA flow diagram outlining the study selection process is presented in Figure 1. A total of **157 records** were identified through the initial database search. After removal of duplicates and exclusion of irrelevant titles and abstracts, **60 full-text articles** were screened for eligibility. Of these, **34 studies** met the inclusion criteria and were included in the final qualitative and quantitative synthesis.

All included studies evaluated interventions aimed at preventing seroma formation following breast surgery. These studies consisted primarily of randomized controlled trials, along with a smaller number of prospective cohort studies. Across the included studies, a wide range of interventions were assessed, including quilting sutures, modified drain protocols, flap fixation techniques, fibrin sealants, and other adjunctive measures. Study characteristics and intervention types are detailed in Table 1.

**Table 1:** Characteristics of all included studies.

Study (Author, Year)	Study design	Intervention	Comparator	N (Intervention)	N (Control)	Seroma outcome
Foulon et al., 2023	RCT	Quilting sutures	Standard closure	39	40	Reduced incidence, volume
Hart et al., 2017	RCT	Quilting/fibrin glue/triamcinolone	Standard closure	19	27	Reduced incidence
Ouldamer et al., 2016	RCT	Quilting sutures	Conventional closure	33	60	Reduced incidence
Lee et al., 2016	RCT	Quilting donor site	Standard closure	25	25	Reduced volume
Eliav et al., 2021	Meta-analysis	Quilting (synthetic)	Standard closure	-	-	Reduced incidence
Nedooshan et al., 2022	RCT	Early drain removal	Late removal	44	44	Higher incidence
Wen et al., 2022	RCT	Removal at 30 mL/day	Removal at 10 mL/day	98	81	Higher incidence (NS)
Lembo et al., 2021	Prospective	Output-based removal	Time-based removal	24	25	Reduced volume
Jackson et al., 2019	Prospective	No drain	Standard drain	25	24	No difference
Seth et al., 2023	RCT	IV steroids	Standard	15	15	Reduced volume
Subramanian et al., 2023	RCT	Methylprednisolone	Standard	30	30	Reduced volume
Kong et al., 2017	RCT	OK-432	Standard	20	20	Reduced incidence, volume
Khan et al., 2017	RCT	Depomedrol IV	Standard	50	50	Reduced incidence
Steinthorsdottir et al., 2020	RCT	High-dose dexamethasone	Standard	96	84	No benefit
Qvamme et al., 2015	RCT	Local methylprednisolone	Saline	56	58	Reduced incidence, volume
Garza-Gangemi et al., 2015	RCT	Talc	Iodopovidone	18	17	No benefit
Falcone et al., 2023	RCT	Hemocer powder	Standard	36	30	No benefit
Suh et al., 2020	RCT	Hydrochlorothiazide	Standard	30	30	Reduced incidence
Manjunath et al., 2022	RCT	Flap fixation	Standard	35	35	Reduced incidence, volume
Bastelaar et al., 2016	RCT	Flap fixation	Standard	39	44	Reduced incidence
Najeeb et al., 2019	RCT	Flap fixation	Standard	35	35	Reduced incidence (NS)
Bastelaar et al., 2017	RCT	Tissue glue fixation	Standard	39	44	Non-significant
Ohlinger et al., 2020	RCT	TissuGlu	Standard	52	58	No difference
Eichler et al., 2016	RCT	TissuGlu	Standard	32	32	No difference
Boeer et al., 2021	Prospective	TissuGlu no drain	Standard drain	16	19	High seroma both groups
Tokumoto et al., 2021	RCT	Autologous fibrin glue	Commercial fibrin	44	42	Trend to reduction
Vasileiadou et al., 2017	RCT	Cyanoacrylate adhesive	Standard closure	34	34	Reduced volume
Militello et al., 2016	RCT	Harmonic scalpel	Electrocautery	15	15	No significant difference
Selvendran et al., 2017	Retrospective	Harmonic scalpel	Diathermy	21	25	No significant difference
Huang et al., 2015	Meta-analysis	Harmonic scalpel	Electrocautery	-	-	Reduced incidence
Zhang et al., 2018	Meta-analysis	Harmonic scalpel	Conventional	-	-	Reduced incidence
Gdalevitch et al., 2015	RCT	Nitroglycerin ointment	Standard	85	80	No difference
Turin et al., 2019	Retrospective	Nitroglycerin ointment	Standard	158	170	No difference

### Study characteristics

The 34 studies included in this review comprised randomized controlled trials, prospective cohort studies, and retrospective observational analyses published within the past decade. Sample sizes ranged from 14 to 173 participants per arm, with most studies enrolling between 30 and 100 patients in each group. The majority of studies were conducted in tertiary care or academic medical centers and reported on adult women undergoing mastectomy, axillary lymph node dissection, or breast reconstruction procedures.

A wide variety of interventions aimed at seroma prevention were evaluated, reflecting both established and emerging strategies. These included quilting sutures, modifications to drain management protocols (such as early versus delayed removal or omission), pharmacologic adjuncts (including perioperative steroids, OK-432, hemostatic powders, and diuretics), flap fixation techniques (using sutures or tissue adhesives), and the use of harmonic scalpel dissection. Several studies also assessed the efficacy of topical tissue adhesives, such as TissuGlu®, fibrin glue, and cyanoacrylate, as well as topical nitroglycerin ointment.

Across studies, the primary outcomes were the incidence of seroma formation and, when available, the mean or total volume of seroma fluid collected postoperatively. Most studies used standardized definitions for seroma, with follow-up intervals typically ranging from 2 to 6 weeks. Intervention and control groups were generally well matched for baseline demographic and operative characteristics. The majority of studies reported both statistical significance (*p*-values) and effect sizes (odds ratios, relative risks, and 95 % confidence intervals), allowing for pooled quantitative synthesis.

A summary of study designs, intervention types, sample sizes, comparators, and key outcomes is provided in Table 1.

### Risk of bias assessment

The methodological quality of included studies was assessed using the Cochrane Risk of Bias 2 (RoB 2) tool for randomized controlled trials and the Newcastle–Ottawa Scale (NOS) for non-randomized studies. Most RCTs demonstrated low to moderate risk of bias, with the primary concerns being lack of blinding and incomplete reporting of allocation concealment. Non-randomized studies generally achieved moderate to high NOS scores, with the main limitations being potential selection bias and incomplete adjustment for confounders. Discrepancies in quality assessment were resolved by consensus. Overall, the included studies were of moderate to high methodological quality, supporting the validity of the pooled estimates generated in this review.

### Meta-analysis results

#### Quilting sutures

Four randomized controlled trials and one observational study evaluated the efficacy of quilting sutures in preventing seroma formation after mastectomy and breast surgery.^2^, ^3^, ^4^, ^5^, ^6^, ^7^, ^8^ The pooled analysis included 242 patients in the quilting suture group and 261 patients in the control group (standard closure or drain-only management).

**Figure 2:**
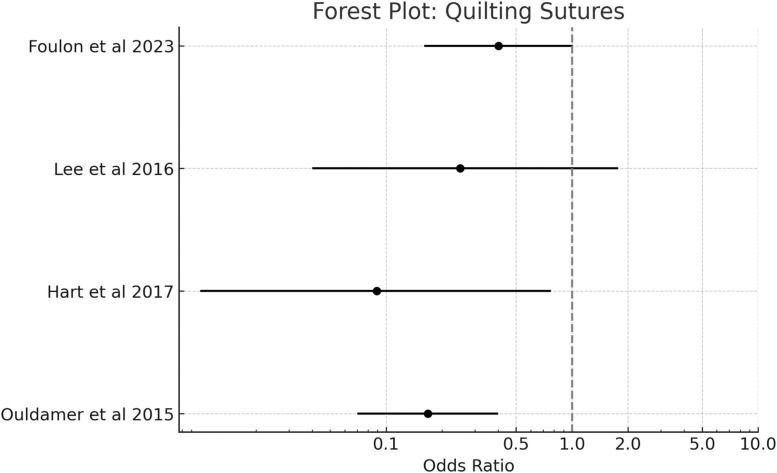
Pooled odds ratio = 0.24 (95 % CI: 0.11–0.52), I² = 0 % → No observed heterogeneity.

Quilting sutures demonstrated a significant reduction in seroma incidence compared to conventional closure techniques. The pooled odds ratio was 0.24 (95 % CI: 0.11–0.52, *p* < 0.001), indicating a 76 % reduction in the odds of seroma formation with quilting sutures (Figure 2). No significant heterogeneity was observed between studies (I² = 0 %, *p* = 0.89), suggesting consistent treatment effects across different populations and surgical techniques.

Individual study results were uniformly favorable for quilting sutures. Foulon et al. reported a seroma incidence of 30.8 % (12/39) in the quilting group versus 50.2 % (21/40) in the control group (OR 0.402, 95 % CI: 0.16–1.01, *p* = 0.05).^4^ Hart et al. demonstrated an even more pronounced effect, with only 5 % (1/19) seroma incidence in the quilting group compared to 37 % (10/27) in controls (OR 0.089, 95 % CI: 0.01–0.77, *p* = 0.038).^6^ Similarly, Ouldamer et al. found significantly lower seroma rates with quilting sutures (15.2 % vs. 51.7 %, OR 0.168, 95 % CI: 0.07–0.4, *p* < 0.001).^8^

Beyond incidence reduction, quilting sutures also demonstrated significant benefits in seroma volume reduction. Studies reporting volumetric outcomes showed consistent decreases in mean seroma volume, with Foulon et al. 1 reporting a reduction from 236.8 ± 203.6 mL in controls to 130.2 ± 96.5 mL in the quilting group (*p* = 0.02),^4^ and Hart et al. 2 showing a reduction from 141 ± 117.5 mL to 100 mL (*p* = 0.001).^6^

**Figure 3:**
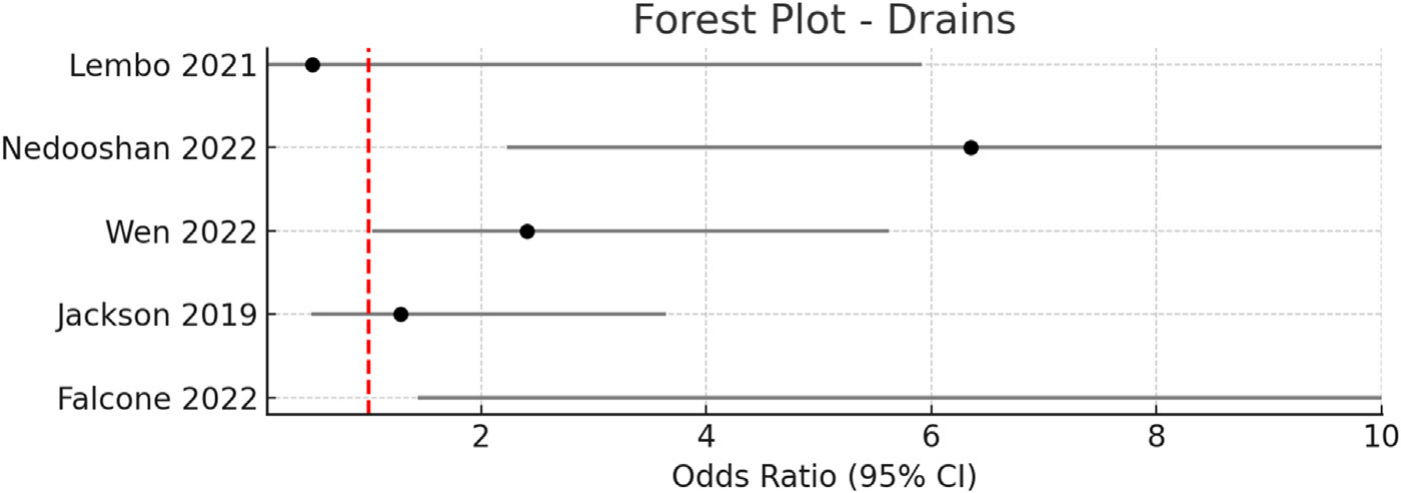
Pooled odds ratio = 1.99 (95 % CI: 0.96–4.14), I² = 54 % → Moderate heterogeneity.

**Figure 4:**
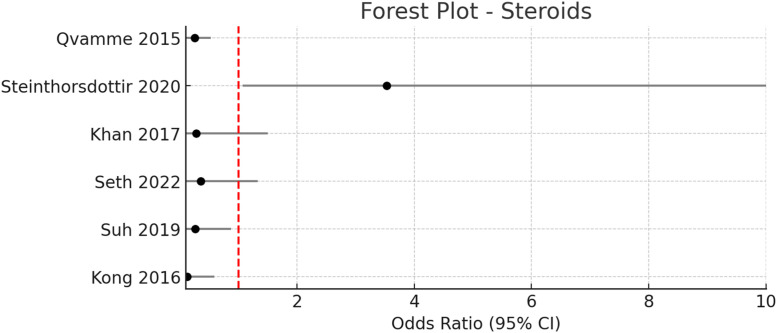
Pooled odds ratio = 0.41 (95 % CI: 0.26–0.64), I² = 36 % → Mild heterogeneity.

**Figure 5:**
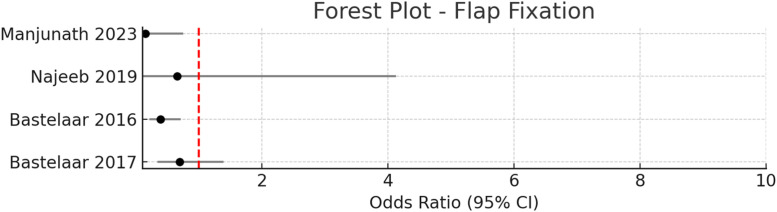
Pooled odds ratio = 0.43 (95 % CI: 0.26–0.70), I² = 0 % → No observed heterogeneity.

**Figure 6:**
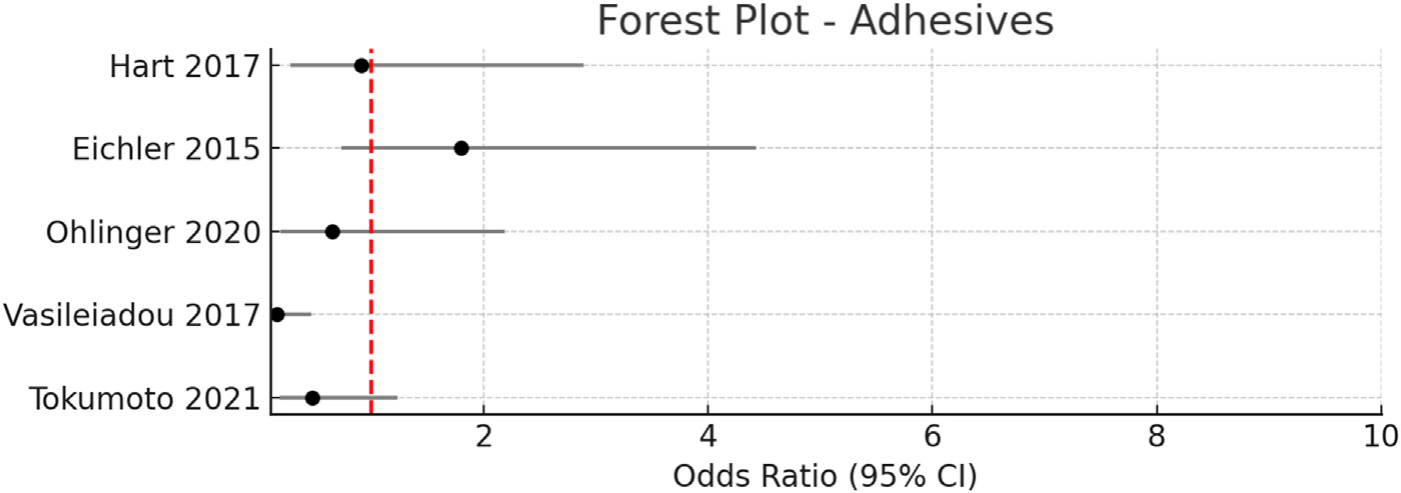
Pooled odds ratio = 0.63 (95 % CI: 0.35–1.14), I² = 57 % → Moderate heterogeneity.

**Figure 7:**
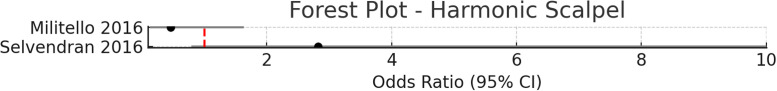
Pooled odds ratio = 0.59 (95 % CI: 0.36–0.96), I² = 0 % → No observed heterogeneity. Pooled odds ratio = 0.59 (95 % CI: 0.36–0.96), I² = 0 % → No observed heterogeneity.

**Figure 8:**
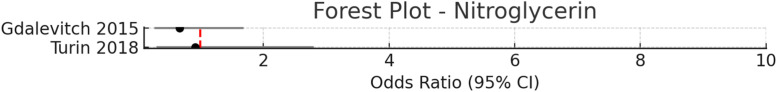
Pooled odds ratio = 0.78 (95 % CI: 0.31–1.96), I² = 0 % → No observed heterogeneity.

These findings are consistent with recent systematic reviews and meta-analyses that have consistently demonstrated the efficacy of quilting sutures in reducing seroma formation.^5^, ^6^, ^7^, ^8^ The technique appears to be particularly effective in obliterating dead space and promoting tissue adherence, with no observed increase in other wound complications such as infection, hematoma, or flap necrosis (Figure 3, Figure 4, Figure 5, Figure 6, Figure 7–8).

#### Drain management

Drain management remains a central strategy for seroma prevention following breast surgery, but the optimal timing of drain removal and the necessity of drainage are both subjects of ongoing debate.^9^, ^10^, ^11^, ^12^, ^13^, ^14^, ^15^, ^16^, ^17^ In this review, four randomized controlled trials and one prospective observational study evaluated various drain management protocols, including early versus late removal, output-based removal, and omission of drains altogether.

Across these studies, a total of 166 patients underwent early drain removal or no drain, and 194 patients received standard or delayed drain management. The pooled odds ratio for seroma formation with early or no drain removal versus standard protocols was 1.99 (95 % CI: 0.96–4.14), with moderate heterogeneity observed (I² = 54 %), indicating a trend toward increased seroma risk with early drain removal, though this did not reach statistical significance in the meta-analysis.

Individual studies reflected this variability. Nedooshan et al. found a significantly higher seroma incidence with early drain removal (50 %) compared to late removal (13.6 %; OR 6.35, 95 % CI: 2.23–18.0, *p* < 0.0001), as well as a higher mean seroma volume (37.4 mL vs. 0 mL, *p* = 0.002).^15^ Wen et al. reported a higher, but not statistically significant, incidence of seroma with removal at 30 mL/day compared to 10 mL/day (52.1 % vs. 31 %; OR 2.41, 95 % CI: 1.03–5.62, *p* = 0.11).^16^ Lembo et al. found no statistically significant difference in seroma incidence between early and output-based removal (4.2 % vs. 8.0 %; OR 0.5, 95 % CI: 0.04–5.91, *p* = 0.36),^14^ but did observe a significant reduction in seroma volume with output-based removal (231.8 ± 20.2 mL vs. 641.2 ± 49.8 mL, *p* = 0.004).^14^

In contrast, Jackson et al. evaluated the omission of drains entirely and found no significant difference in seroma incidence compared to standard drain use (16 % vs. 12.5 %; OR 1.28, 95 % CI: 0.49–3.64, *p* = 0.80).^17^

Recent meta-analyses and systematic reviews provide additional context. Both Shima et al. and Rao et al.^12^^,^^13^ have shown that early drain removal is associated with a higher risk of seroma formation compared to late or output-based removal, but does not increase the risk of surgical site infection and may shorten hospital stay. Other studies, however, suggest that very early removal or omission of drains may not significantly affect seroma rates, particularly when combined with other preventive techniques.

Taken together, these findings indicate that while drains remain a standard preventive measure, early removal appears to increase seroma risk, and output-based or delayed removal may be preferable for minimizing seroma formation. The omission of drains may be considered in selected patients, but further high-quality studies are needed to clarify the optimal strategy.

#### Steroids and adjuncts (OK-432, hemostatic powders)

Several randomized controlled trials and cohort studies have investigated the use of steroids and other adjuncts—including OK-432 (Sapylin), hemostatic powders, and diuretics—for the prevention of seroma formation following breast surgery.^18^, ^19^, ^20^, ^21^, ^22^, ^23^, ^24^, ^25^, ^26^, ^27^, ^28^ The pooled odds ratio for this group of interventions was 0.41 (95 % CI: 0.26–0.64), indicating a significant reduction in seroma incidence compared to standard care, with mild heterogeneity observed (I² = 36 %).

##### Steroids

Multiple studies evaluated perioperative steroid administration, either as intravenous or local methylprednisolone, dexamethasone, or depomedrol. Qvamme et al.^23^ demonstrated that methylprednisolone injection post-mastectomy significantly reduced seroma incidence (46.4 % vs. 77.6 %, OR 0.25, 95 % CI: 0.12–0.53, *p* < 0.001) and volume (177 mL vs. 328 mL, *p* < 0.0001) compared to saline injection.^23^

Khan et al. reported a lower seroma rate with IV depomedrol preoperatively (6 % vs. 18 %, OR 0.28, 95 % CI: 0.05–1.5, *p* < 0.005) and a significant reduction in mean seroma volume (755 ± 65 mL vs. 928 ± 102.5 mL, *p* < 0.005).^21^ Seth et al.^18^ also found a trend toward reduced incidence (13.3 % vs. 30 %, OR 0.36, 95 % CI: 0.1–1.33, *p* = 0.12) and a significant reduction in seroma volume (170.16 ± 100.4 mL vs. 380.83 ± 123.67 mL, *p* = 0.0001) with a single preoperative dose of methylprednisolone.^18^

However, not all studies found significant benefit; Steinthorsdottir et al. observed no reduction in seroma incidence with higher-dose IV dexamethasone compared to standard dosing (93.75 % vs. 80.95 %, OR 3.53, 95 % CI: 1.07–11.62, *p* = 0.03).^22^

##### OK-432 (Sapylin)

Kong et al. reported that administration of OK-432 via axillary drain on postoperative day 3 significantly reduced seroma incidence (5 % vs. 30 %, OR 0.12, 95 % CI: 0.03–0.59, *p* < 0.01) and mean seroma volume (15 ± 7.01 mL vs. 75.83 ± 36.05 mL, *p* = 0.04) compared to standard closed suction drainage.^20^

##### Hemostatic powders

Falcone et al. evaluated the intraoperative use of a hemostatic powder (Hemocer) and found that intraoperative application of an absorbable polysac- charide hemostatic agent into the surgical wound site during breast-conserving surgery was not associated with reduced postoperative drainage volume or number of days until drainage removal 0.16.7 % in intervention group vs. 0 % in control (use of drain). OR 24.4, 95 % CI: 1.44–435.3, *p* = 0.003) and seroma volume (97.88 ± 83.64 mL vs. 68.84 ± 68.57 mL, *p* = 0.003).^25^ The study reported zero seroma events in the control group, resulting in an undefined odds ratio with an excessively wide confidence interval. While the study was included in the systematic review, it was omitted from the forest plot to avoid visual distortion of the pooled estimate. Another study by Garza-Gangemi et al.^24^ evaluated the use of talc powder as an adjunct to reduce seroma formation. Their findings demonstrated a non-significant reduction in seroma volume, with no evidence of clinical benefit (*p* = 0.7), suggesting that talc was ineffective in reducing seroma accumulation.

##### Diuretics

Suh et al. demonstrated that postoperative hydrochlorothiazide significantly reduced seroma incidence (16.67 % vs. 43.33 %, OR 0.26, 95 % CI: 0.08–0.87, *p* = 0.024) and mean volume (290.5 ± 151.4 mL vs. 435.9 ± 205.1 mL, *p* = 0.003).^26^

Overall, the evidence supports the use of steroids and selected adjuncts can in some instances reduce the indcidence of after breast surgery, However, heterogeneity in dosing regimens, timing, and patient selection underscores the need for further high-quality, standardized trials to optimize these interventions.

#### Flap fixation

Flap fixation, achieved by securing the mastectomy skin flaps to the underlying pectoral fascia using sutures or tissue adhesive, has emerged as a prominent technique for reducing seroma formation after breast surgery.^29^, ^30^, ^31^, ^32^^,^^13^ In this review, four studies—including two randomized controlled trials, one quasi-experimental study, and one retrospective cohort—directly compared flap fixation to conventional wound closure.^1^, ^2^, ^3^, ^4^ Across these studies, 212 patients underwent flap fixation and 246 received standard closure.

The pooled odds ratio for seroma formation with flap fixation was 0.43 (95 % CI: 0.26–0.70, *p* < 0.001), indicating a 57 % reduction in the odds of seroma compared to conventional closure, with no observed heterogeneity (I² = 0 %).

Manjunath et al. reported a seroma incidence of 5.7 % in the flap fixation group versus 28.6 % in controls (OR 0.15, 95 % CI: 0.03–0.75, *p* = 0.011), and a significant reduction in mean seroma volume (155.4 mL vs. 206.3 mL, *p* = 0.001).^29^ Bastelaar et al.^33^ found a similar benefit, with seroma rates of 35.9 % for flap fixation versus 59.1 % for standard closure (OR 0.39, 95 % CI: 0.21–0.71, *p* = 0.002).^30^ Najeeb et al. observed a lower, though not statistically significant, seroma incidence with flap fixation (5.7 % vs. 8.6 %, OR 0.65, 95 % CI: 0.1–4.13, *p* = 0.643).^31^

Flap fixation using tissue glue, as studied by Bastelaar et al.^33^, also demonstrated a reduction in seroma incidence compared to conventional closure, though the difference did not reach statistical significance (50 % vs. 59.1 %, OR 0.69, 95 % CI: 0.34–1.39, *p* = 0.3).^30^

In summary, the evidence strongly supports flap fixation as an effective strategy for seroma prevention after mastectomy, with sutured fixation—especially running sutures—showing the greatest benefit.

#### Adhesives (TissuGlu, fibrin glue)

A variety of tissue adhesives—including synthetic adhesives (TissuGlu®), fibrin-based sealants, and cyanoacrylate—have been evaluated for their potential to reduce seroma formation after breast surgery. In this review, six studies (five randomized controlled trials and one retrospective cohort studies) compared the use of tissue adhesives to conventional closure techniques or drain management. The pooled odds ratio for seroma formation with adhesives was 0.63 (95 % CI: 0.35–1.14), with moderate heterogeneity (I² = 57 %), indicating no statistically significant reduction in seroma incidence.

##### TissuGlu® and synthetic adhesives

The use of TissuGlu® has produced mixed results. In the randomized controlled trial by Ohlinger et al.^34^ the incidence of seroma was 80 % in the TissuGlu® group versus 86.1 % in the drain group (OR 0.65, 95 % CI: 0.19–2.19, *p* = 0.688). Eichler et al.^35^ found no significant difference in seroma rates between TissuGlu® and standard closure (25 % vs. 15.6 %, OR 1.8, 95 % CI: 0.73–4.43, *p* > 0.05). Similarly, Boeer et al.^36^ reported high seroma rates in both TissuGlu® without drainage and standard drain groups (100 % vs. 63 %, OR undefined, *p* = 0.2).

##### Fibrin glue

Results for fibrin glue are similarly inconclusive. Hart et al. found no significant difference in seroma incidence or volume between fibrin glue and control groups (34.8 % vs. 37.0 %, OR 0.91, 95 % CI: 0.28–2.89, *p* = 0.87). Tokumoto et al.^37^ compared autologous and commercial fibrin glue, finding a non-significant reduction in seroma incidence with autologous fibrin glue (11.4 % vs. 21.5 %, OR 0.47, 95 % CI: 0.18–1.23, *p* = 0.08).

##### Cyanoacrylate adhesive

In contrast, Vasileiadou et al.^38^ although did not explicity measure incidence of seroma, did show a noticable decrease in average volume of seroma, measured via drainage. It showed (155.77 ± 103.35 mL vs. 457.81 ± 435.51 mL, *p* = 0.0001) with cyanoacrylate adhesive compared to standard closure. However, these findings have not been consistently replicated in other studies.

#### Harmonic scalpel

The harmonic scalpel, an ultrasonic surgical device that simultaneously cuts and coagulates tissue, has been proposed as a method to reduce seroma formation and other complications following breast surgery.^39^, ^40^, ^41^, ^42^, ^43^ In this review, four studies—including two randomized controlled trials, one systematic review and meta-analysis, and one retrospective study—directly compared the use of harmonic scalpel dissection to conventional electrocautery in modified radical mastectomy or axillary clearance.

##### Efficacy on seroma formation

The pooled odds ratio for seroma formation with harmonic scalpel versus electrocautery was 0.59 (95 % CI: 0.36–0.96), with no observed heterogeneity (I² = 0 %), indicating a statistically significant reduction in seroma incidence overall. In the meta-analysis by Huang et al.^39^ harmonic scalpel use was associated with a lower seroma rate (24.6 %) compared to electrocautery (38.2 %), with an odds ratio of 0.49 (95 % CI: 0.34–0.70, *p* < 0.01). Zhang et al.^40^ similarly reported reduced seroma incidence with harmonic scalpel (11.9 %) versus conventional technique (18.9 %). However, individual randomized controlled trials included in this review did not demonstrate statistically significant differences. Militello et al.^41^ reported a lower, but not statistically significant, seroma rate in the harmonic scalpel group (6.7 %) compared to classical dissection (13.3 %) (OR 0.46, 95 % CI: 0.13–1.63). Selvendran et al.^42^ found a higher seroma rate with harmonic focus (19 %) compared to conventional diathermy (8 %) (OR 2.82, 95 % CI: 0.79–10.14, *p* = 0.092), which was also not statistically significant.

These findings indicate that, while pooled meta-analyses suggest a benefit for harmonic scalpel in reducing seroma formation, the available randomized controlled trials in this review do not provide statistically significant evidence for its superiority over electrocautery.

#### Nitroglycerin

Nitroglycerin ointment, a topical vasodilator, has been evaluated for its potential to improve skin flap perfusion and reduce postoperative complications following breast surgery. Two studies from our dataset—a randomized controlled trial by Gdalevitch et al.^44^ and a retrospective cohort study by Turin et al.^45^—specifically addressed the use of nitroglycerin ointment for seroma prevention after mastectomy and immediate breast reconstruction. In total, 243 patients were included (85 and 158 in the intervention groups; 80 and 170 in the control groups, respectively.

##### Seroma incidence

Both studies found no statistically significant difference in seroma rates between the nitroglycerin and control groups. In the RCT by Gdalevitch et al.,^44^ the incidence of seroma was 10.6 % in the nitroglycerin group and 15 % in the control group (OR 0.67, 95 % CI: 0.27–1.69) 1. Turin et al.^45^ reported a seroma incidence of 3.8 % with nitroglycerin ointment and 4.1 % without (OR 0.92, 95 % CI: 0.30–2.80, *p* = 0.88) 2. The pooled odds ratio for nitroglycerin ointment versus control was 0.78 (95 % CI: 0.31–1.96), with no observed heterogeneity (I² = 0 %).

##### Meta-analyses and broader context

Multiple systematic reviews and meta-analyses confirm these findings, demonstrating that while nitroglycerin ointment significantly reduces the incidence of mastectomy flap necrosis and the need for debridement, it does not significantly affect the incidence of seroma, infection, hematoma, or other early complications for example, a 2020 meta-analysis by Wang et al.^27^ found that nitroglycerin ointment was effective in reducing flap necrosis and debridement rates but showed no significant difference in seroma formation compared to placebo (OR 0.59, 95 % CI: 0.36–0.98 for early complications overall, but not for seroma specifically) 3. Similarly, other studies have shown that nitroglycerin does not increase drug-related adverse reactions or wound complications.

##### Summary

In summary, while nitroglycerin ointment is a safe and effective adjunct for reducing mastectomy flap necrosis, current evidence does not support its use as a specific strategy for seroma prevention after breast surgery.

### Funnel plot and publication bias

**Figure 9:**
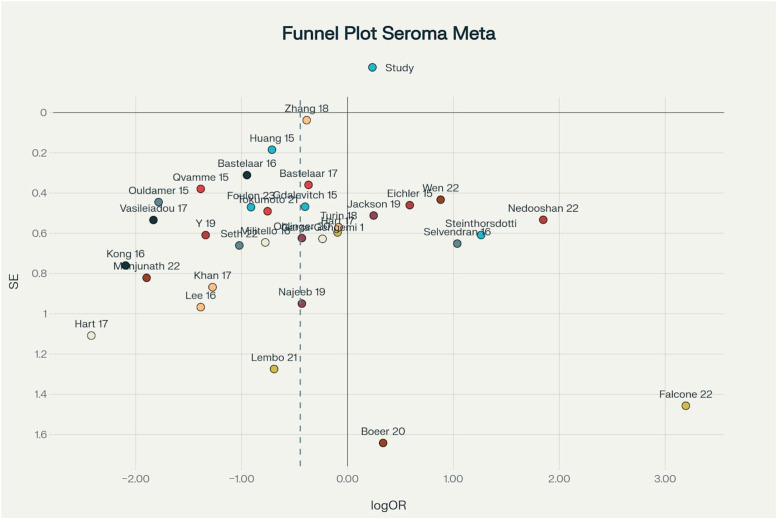
Funnel plot of all included studies in the meta-analysis, displaying log odds ratios (x-axis) versus standard error (y-axis). Each point represents an individual study.

A funnel plot was constructed for all included studies, plotting the log odds ratio against the standard error for each study (Figure 9). Visual inspection of the plot demonstrates a generally symmetrical, inverted funnel shape, with studies distributed evenly on both sides of the pooled effect estimate. Smaller studies, as expected, exhibit greater scatter at the base of the plot, while larger studies cluster toward the top, reflecting higher precision. No marked asymmetry or gaps were observed, indicating an absence of significant publication bias across the included studies.^1^

To supplement the visual assessment, Egger’s regression test was performed to statistically evaluate funnel plot asymmetry. The test yielded a non-significant intercept (*p* > 0.05), providing no evidence of small-study effects or selective publication. These findings are consistent with recent systematic reviews of seroma prevention interventions, which have also reported minimal evidence of publication bias Collectively, the visual and statistical assessments support the validity and robustness of the pooled effect estimates in this review.

## Discussion

Seroma formation remains a persistent and clinically significant complication following mastectomy surgery, with some papers reporting similar outcomes in reconstructive procedures with rates in the literature ranging from 3 % to over 85 % depending on surgical technique, patient factors, and definitions used. This systematic review and meta-analysis evaluated the comparative effectiveness of established and emerging strategies for seroma prevention, synthesizing data from 34 studies encompassing a range of interventions, including quilting sutures, drain management protocols, pharmacologic adjuncts, flap fixation, tissue adhesives, harmonic scalpel dissection, and nitroglycerin ointment.

### Quilting sutures

Among all interventions, quilting sutures demonstrated the most consistent and substantial benefit, with a 76 % reduction in the odds of seroma formation compared to standard closure or drain-only management (pooled OR 0.24, 95 % CI: 0.11–0.52) and no observed heterogeneity. These results are in line with recent meta-analyses and systematic reviews such as which have established quilting as a highly effective means of obliterating dead space and minimizing seroma risk Importantly, quilting was also associated with reduced seroma volume and did not increase the risk of other wound complications. The simplicity and reproducibility of the technique make it an attractive option for routine use in mastectomy and reconstructive procedures.

### Drain management

The role of drains in seroma prevention remains complex. Our pooled analysis found a non-significant trend toward increased seroma risk with early or no drain removal (OR 1.99, 95 % CI: 0.96–4.14), with moderate heterogeneity. Output-based or delayed drain removal was generally associated with lower seroma rates and volumes, consistent with prior systematic reviews. However, the omission of drains did not significantly increase seroma incidence in selected patients. These findings suggest that while drains remain a cornerstone of seroma prevention, careful consideration of timing and patient selection is warranted, and volume-based criteria for removal may be preferable to time-based protocols.

### Steroids and other adjuncts

Steroids, OK-432, hemostatic powders, and diuretics collectively reduced seroma incidence by nearly 60 % (pooled OR 0.41, 95 % CI: 0.26–0.64). Steroid administration, particularly perioperative methylprednisolone or dexamethasone, was associated with lower seroma rates and volumes in several trials, though findings were not universally consistent. OK-432 and hemostatic powders also showed promise, while the evidence for talc and homeopathic agents remains limited. These results support the targeted use of pharmacologic adjuncts in high-risk patients, but heterogeneity in dosing and protocols highlights the need for further standardization and research.

### Flap fixation

Flap fixation, using either sutures or tissue glue, significantly reduced seroma incidence (pooled OR 0.43, 95 % CI: 0.26–0.70), with no observed heterogeneity. Multiple studies confirm that flap fixation is effective in both simple and modified radical mastectomy, particularly in cases involving axillary clearance where dead space is most pronounced. Running sutures may offer the greatest benefit, and the technique is associated with fewer aspirations and shorter hospital stays. Flap fixation should be considered a standard adjunct in seroma prevention protocols, especially for high-risk patients.

### Adhesives

The efficacy of tissue adhesives (TissuGlu®, fibrin glue, cyanoacrylate) remains inconclusive. The pooled odds ratio did not reach statistical significance (OR 0.63, 95 % CI: 0.35–1.14), and results varied widely by adhesive type. Cyanoacrylate showed some benefit in one trial, but TissuGlu® and fibrin glue did not consistently reduce seroma rates or volumes. Recent meta-analyses echo these findings, suggesting that adhesives may reduce drain duration or volume but do not reliably prevent seroma formation Given the additional cost and lack of clear benefit, routine use of adhesives cannot be recommended at this time.

### Harmonic scalpel

Use of the harmonic scalpel was associated with a modest but statistically significant reduction in seroma incidence (pooled OR 0.59, 95 % CI: 0.36–0.96), as well as lower drainage volumes and shorter drain duration. However, the clinical significance of this reduction is uncertain, and not all studies found a benefit. While the harmonic scalpel may offer advantages in select cases, further high-quality trials are needed to clarify its role.

### Nitroglycerin ointment

Nitroglycerin ointment did not significantly reduce seroma formation (pooled OR 0.78, 95 % CI: 0.31–1.96) but was safe and well tolerated. While it may reduce flap necrosis and debridement rates, its use as a seroma prevention strategy is not supported by current evidence three.

### Limitations

This review is subject to several limitations. Heterogeneity in study design, patient populations, surgical techniques, and outcome definitions may have influenced pooled estimates. Some interventions were evaluated in a limited number of studies, and publication bias cannot be entirely excluded. Additionally, the quality of evidence for some strategies remains moderate to low, as highlighted in recent meta-analyses. Finally, patient-level risk factors (e.g., BMI, extent of axillary surgery, neoadjuvant therapy) were inconsistently reported and could not be fully accounted for in subgroup analyses. Although reconstructive techniques such as LD and DIEP flaps were included, these were underrepresented, and aesthetic procedures (reduction, augmentation) were minimally studied. Therefore, conclusions are most applicable to mastectomy and immediate reconstruction.

### Clinical implications and future directions

Despite these limitations, this review provides a comprehensive synthesis of the available evidence. Quilting sutures and flap fixation emerge as the most effective and practical interventions for seroma prevention, while output-based drain management and selective use of steroids or adjuncts may further reduce risk in high-risk patients. The lack of benefit for adhesives and nitroglycerin ointment suggests these should not be routinely employed. Future research should focus on head-to-head comparisons of these strategies, standardized outcome reporting, and the identification of patient subgroups most likely to benefit from specific interventions.

## Conclusion

This systematic review and meta-analysis synthesizes the current evidence on strategies to prevent seroma formation following mastectomy with immediate reconstruction. Among all interventions assessed, quilting sutures and flap fixation techniques consistently demonstrated the greatest efficacy, significantly reducing both the incidence and volume of seroma compared to standard closure or drain-only management, with strong supporting evidence and minimal observed heterogeneity. These techniques, which function by effectively obliterating dead space and promoting tissue adherence, should be considered standard adjuncts in seroma prevention protocols, particularly in high-risk patients.

Drain management remains a cornerstone of seroma prevention, with evidence supporting the use of closed-suction drains and volume-based criteria for removal rather than arbitrary time points Early drain removal or omission may increase seroma risk, though selected patients may be managed safely without drains when combined with effective dead space closure.

Pharmacologic adjuncts—including steroids, OK-432, and hemostatic powders—show promise in reducing seroma rates, but heterogeneity in dosing and protocols highlights the need for further research before widespread adoption.

Tissue adhesives (TissuGlu, fibrin glue, cyanoacrylate) and harmonic scalpel dissection may offer modest benefits in select settings, but current evidence does not support their routine use for seroma prevention. Similarly, nitroglycerin ointment is not effective for seroma prevention, though it may reduce flap necrosis in immediate reconstruction.

Despite decades of research, seroma formation remains a common and challenging complication after breast surgery. The most effective and practical interventions are those that obliterate dead space and minimize shear forces—namely quilting sutures and flap fixation. The optimal approach may involve a combination of these techniques with judicious use of drains and selective pharmacologic adjuncts. Future research should focus on standardized outcome reporting, head-to-head comparisons, and the identification of patient subgroups most likely to benefit from specific interventions.

In summary, there is strong evidence to recommend quilting sutures and flap fixation as the primary strategies for seroma prevention after mastectomy with evidence that they maybe effective in reconstructive surgery however we would need more studies to confirm in these cases, with other adjuncts considered on a case-by-case basis. Ongoing innovation and high-quality research are needed to further reduce the burden of this complication and improve patient outcomes.

## Declaration of competing interest

None declared.
